# Defining a mission-based method to determine a HEMS unit’s actual service area

**DOI:** 10.1186/s13049-019-0640-4

**Published:** 2019-07-01

**Authors:** Jukka Pappinen, Anna Olkinuora, Päivi Laukkanen-Nevala

**Affiliations:** 1 FinnHEMS Research and Development Unit, Lentäjäntie 3, FI-01530 Vantaa, Finland; 2 University of Eastern Finland, Faculty of Health Sciences, P.O. Box 1627, FI-70211 Kuopio, Finland

**Keywords:** Emergency medical services, Geographic information systems, HEMS

## Abstract

**Background:**

Geographical service areas are used as descriptive system indicators in Emergency Medical Service (EMS) related studies and reporting templates. The actual service area may differ significantly from administrative areas; this may lead to inaccuracy in determining indicator values, such as population or mission density, thus making it biased when comparing results between different areas and organizations.

The aim of this study was to introduce a univocal, repeatable and easily adaptable method to determine the actual service area of a helicopter emergency medical service (HEMS) unit for statistical, quality measurement and research purposes using widely available geographical information (GIS) and statistical analysis tools.

**Methods:**

The method was first tested with Tampere HEMS unit. All accepted missions in 2017 were extracted from FinnHEMS database (FHDB). We calculated distance from HEMS base to each accepted mission location. Missions were reordered based on the distance and 99th and 95th percentiles were calculated for mission distances. Convex hulls including 100, 99 and 95% of the missions, and the population and area covered by these missions, were then calculated. The method was repeated for all Finnish HEMS bases.

**Results:**

Approximately 90% of Tampere HEMS unit’s accepted missions took place within 100 km from the base. 10.9% of the missions occurred outside of the administrative service area. 95% convex hull areas are most in line with the everyday experience of where the units actually operate. In Tampere, the 95% convex hull area corresponds to 76,5% of the administrative area’s population and to 89,8% of its area. Calculating the 95% convex hull areas for all Finnish HEMS units results in service areas that overlap at some points, and some areas of the country fall outside of all HEMS service areas.

**Conclusions:**

Administrative areas do not correspond to the actual service areas of HEMS units. The service area of a HEMS unit defined by administrative boundaries may differ significantly from actual operations. Using historical mission data to create a convex hull that incorporates mission locations could offer a standardized and comparable solution for determining actual HEMS unit service areas, which can be used for statistical comparison, quality measurement and system development.

## Introduction

Geographical service areas, and the populations within them, are used as descriptive system indicators in various Emergency Medical Service (EMS) related studies and reporting templates [1]. While these values are used to calculate indicators, such as population or mission density, there is currently no standardized method to consistently determine the boundaries of a service area. This makes it difficult for different areas and organizations to compare results.

An actual service area may be significantly smaller or larger than the area that is defined by administrative, provincial or municipal boundaries, and this is especially true for helicopter-based EMS (HEMS). A very large body of water or a stretch of uninhabited wilderness may create a statistical bias by decreasing spatial density indicator values, causing the effective area of operations to appear remarkably smaller than the administrative area. Conversely, a HEMS unit may also have a significant number of missions outside of its administrative service area, either intentionally or not. Actual service areas of HEMS bases may e.g. overlap due to mutual aid or joint responsibility arrangements.

In Finland, HEMS units operate almost only primary missions and they are dispatched by dispatch centres. HEMS operations are the responsibility of tertiary (university) hospitals which organize the service within their legally obligated coordination area. Typically, that area includes 2–4 secondary hospital districts. The geographical shape of these areas often does not fit well into the actual flight range of the HEMS bases, thus resulting in frequent HEMS responses to certain neighbouring areas as well. Nonetheless, population and area statistics are unrealistically calculated by administrative areas.

In this study we aim to introduce a univocal, repeatable and easily adaptable method, based on historical mission data, to determine the actual service area of a HEMS unit using widely available geographical information (GIS) and statistical analysis tools. The use of this method would improve the comparability of HEMS services in statistics, quality measurement and research, both nationally and internationally, and it could also be utilized in the planning and development of HEMS systems.

## Methods

The authors initiated a working group within the FinnHEMS Research and Development Unit. The FinnHEMS base near Tampere, Finland, was selected as its focus, due to the base’s wide geographic operating area, which includes several hospital districts. We used the FinnHEMS mission database (FHDB) to compile a list of the missions accepted by the Tampere HEMS unit in 2017. Missions that were denied were excluded from the list, however, missions that were cancelled after departure were included. Based on mission data in 2017, the main reasons for denying missions were overlapping missions (36.3%) and weather (28.3%).

First, we determined the exact geographical location for each mission by using the commercial Google Maps application programming interface (API) to geocode the mission addresses. The query program was written by the correspondent author, using Microsoft Excel and the Visual Basic for Applications (VBA) programming environment. Although many HEMS services routinely collect exact mission locations, at the time, FinnHEMS did not save the exact mission coordinates to the mission database for further use.

MapInfo 15 GIS-software (MI) was used to map the geocoded mission coordinates and to calculate the distances from the HEMS base to each mission location. The results were then analysed with SPSS 25 statistical software. Missions were reordered based on their distance from the HEMS base and 99th and 95th percentiles were calculated for mission distances.

A convex hull is the smallest geometric shape which contains a predetermined set of points, in this case mission locations. A non-mathematical visualization would be a shape formed by a rubber band which is stretched over the most extreme points of the point set [2]. MI was then used to calculate a convex hull that included 100, 99 and 95% of the missions. Last, we calculated the population and area covered by these missions.

This method was repeated for all Finnish HEMS bases to calculate the convex hull area that covered 95% of their accepted missions.

## Results

In 2017, the Tampere HEMS unit accepted a total of 2560 missions. We were able to determine at least an approximate location for each of those missions (Table 1).

**Table 1:** Geocoding accuracy of Tampere HEMS unit’s accepted missions in 2017, using Google Maps Geocoding API

Geocoding accuracy	*N*	%
Rooftop (middle of the building)	1711	66.9
Geometric centre of the road	679	26.5
Approximate ^a^	118	4.6
Range interpolated	52	2.0
TOTAL	2560	100.0

^a^ Google Maps does not provide precise estimation for approximated locations

**Fig. 1:**
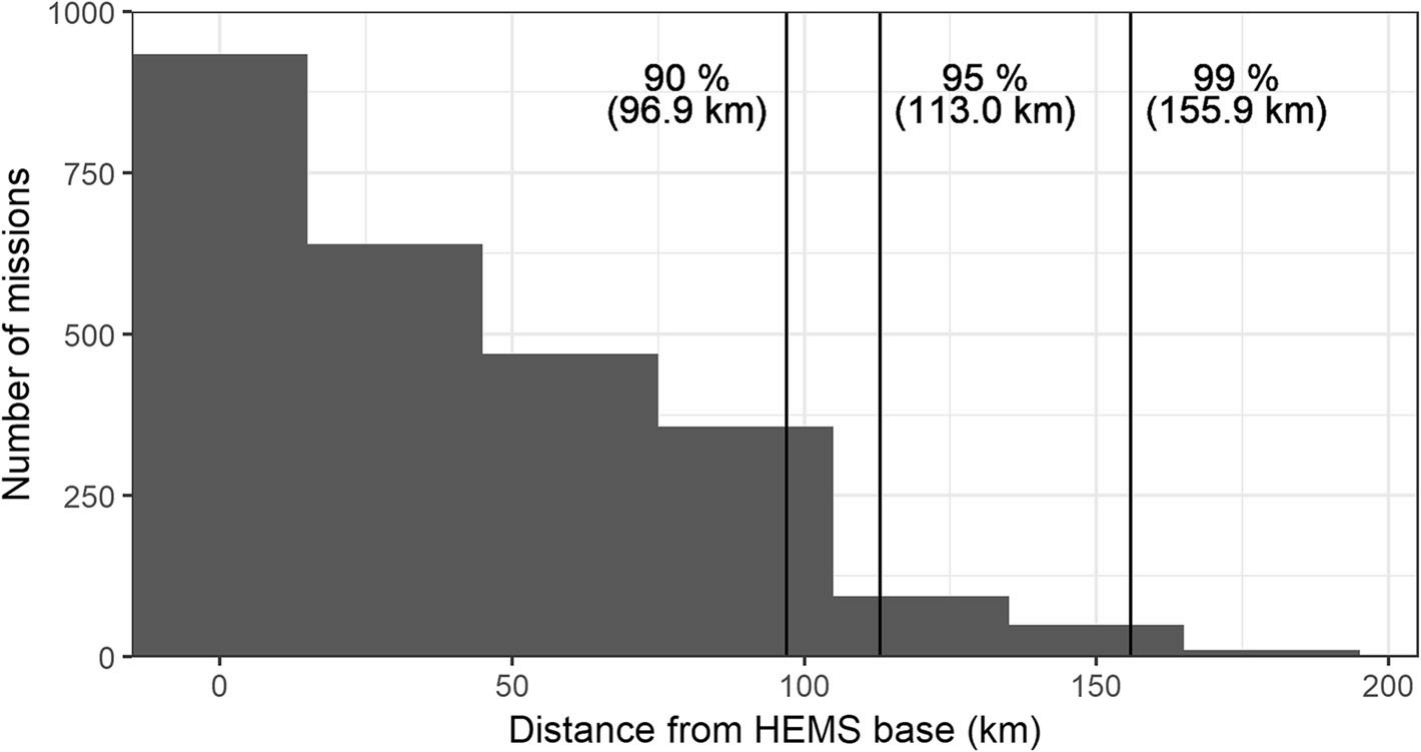
Distances from Tampere HEMS base to accepted mission locations in 2017. Reference lines represent 99, 95 and 90% percentiles

Approximately 90% of the missions took place within a 100 km range of the base (Fig. 1). The histogram shows the typical mission distribution around a major city. The number of missions decreases by distance, with a significant drop after 100 km. Most missions (1913, 74.3%) took place within the Pirkanmaa hospital district, around the city of Tampere, and 2282 (89.1%) missions occurred within the Tampere University Hospital coordination area. 278 (10.9%) missions occurred outside of the administrative coordination area.

**Fig. 2:**
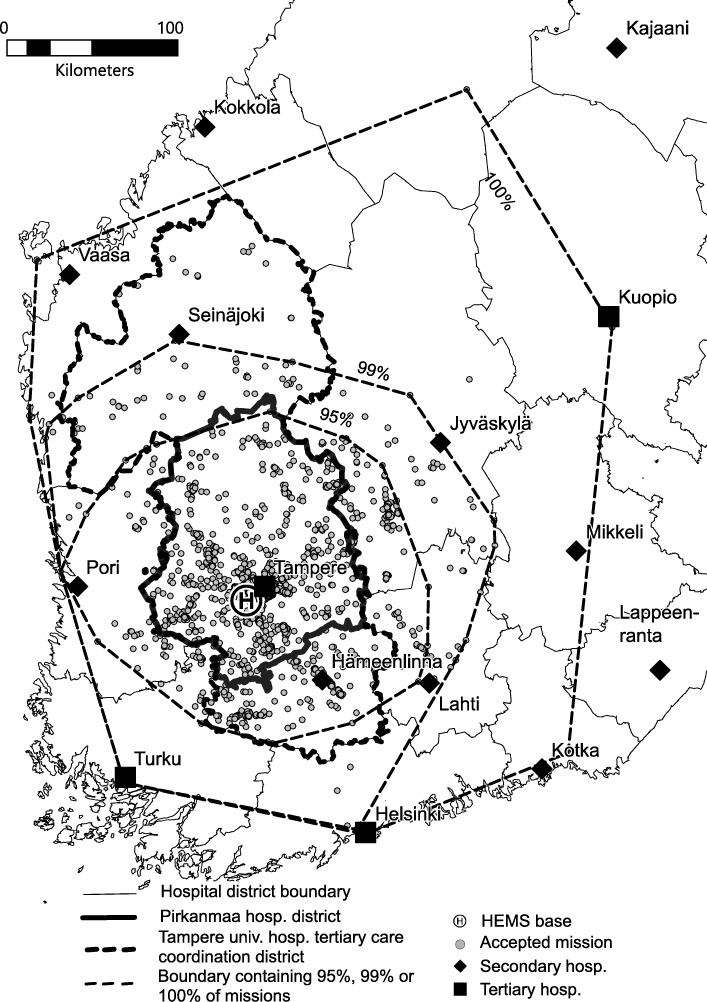
Accepted HEMS missions from Tampere base in 2017 with administrative and convex hull-based service areas. The map contains data from the National Land Survey of Finland Topographic Database 1/2018

Population and service area determined by administrative boundaries and convex hull-based areas with population data are presented in Table 2. Tampere University Hospital is a secondary care provider in the Pirkanmaa Hospital District but has responsibility for coordinating EMS and arranging HEMS operations for two other hospital districts. The population within the convex hull-based area that represents 95% of the missions corresponds to 76.5% of Tampere University Hospital administrative coordination area’s total population and 89.8% of the area. For 99% of the missions, the convex hull-based service area is 63.9% larger than the administrative area, with 80.6% larger population than in the administrative area. The population densities for the area are 32.5 inhabitants/km^2^ for the Tampere University Hospital administrative area, 27.8 inhabitants/km^2^ for the 95% convex hull and 35.9 inhabitants/km^2^ for the 99% convex hull. Based on these observations, it is obvious that the administrative areas do not correspond to the actual service areas or to the populations covered by Tampere HEMS unit’s missions. This is further illustrated in Fig. 2.

**Table 2:** Population and area of administrative and convex hull-based service areas

Area description		Population	Area (km^2^)
Administrative areas	Tampere Univ. Hospital area	1,111,487	34,110
	Pirkanmaa Hospital District	526,941	14,160
Convex hull-based areas	100% convex hull	3,464,322	110,800
	99% convex hull	2,007,062	55,890
	95% convex hull	850,558	30,620

**Fig. 3:**
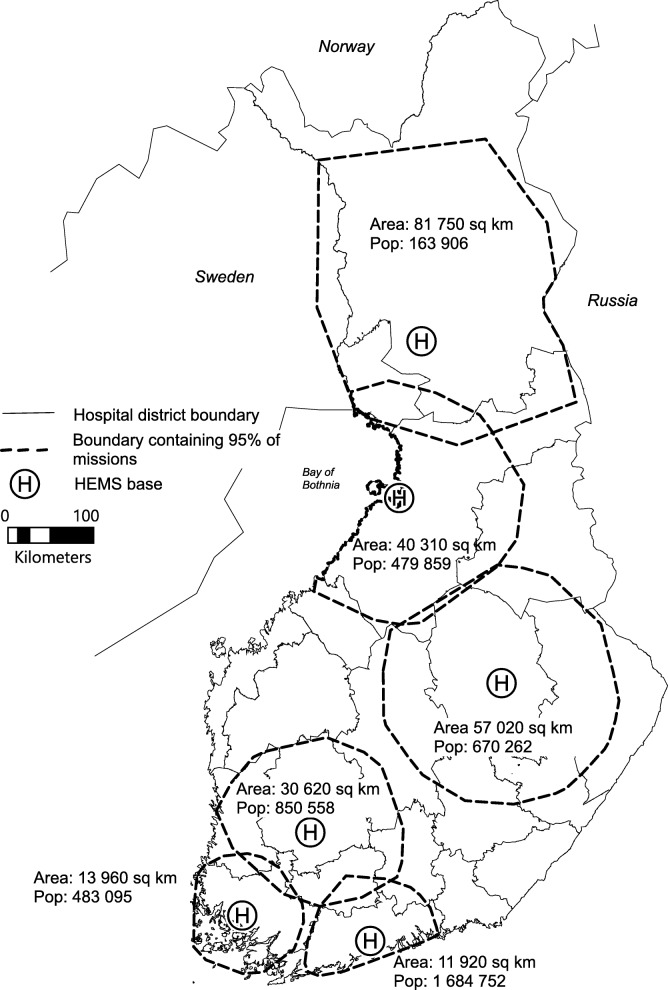
Convex hull-based service areas containing 95% of accepted missions in 2017 for all Finnish HEMS bases. The map contains data from the National Land Survey of Finland Topographic Database 1/2018

Figure 3 presents the convex hull-based service areas of all HEMS bases in Finland, covering 95% of the HEMS unit’s accepted missions. The 95% cut point produces areas which are most in line with the everyday experience of the area where the units actually operate. The resulting service areas overlap at some points and some areas of the country fall outside of all HEMS service areas.

## Discussion

There were two main findings of this study. First, administrative boundaries correspond poorly to the actual mission-based service area of a HEMS unit, thus leading to misleading conclusions, especially when comparing service providers and HEMS systems. Second, using historical mission data and the convex hull-based method could offer a standard solution for defining a service area and its population as a system indicator.

System-specific quality indicators (QIs) for physician-staffed emergency medical services (P-EMS), which were developed by an expert consensus panel, included two indicators that are based on service area: the number of P-EMS units per 100,000 inhabitants and the number of P-EMS units per km^2^ in the area covered by the service [3]. To create comparable results based on these indicators, the definition of service area should be standardized, as it largely determines the result.

Further, time variables have traditionally played an important role when setting targets and measuring EMS quality [4, 5], but they are, at the same time, dependent on the distances in which the unit has to operate. Based on a systematic literature review of quality measurement in physician-staffed emergency medical services, response time was identified as one of the four most widely used QIs; mission duration (measured as the time from alarm to patient handover or the time from arrival at patient until hospital admission) was also mentioned several times [6]. However, if these QIs are to be used for comparing services and setting targets, measuring the mere number of minutes is a poor descriptor of quality as it is mainly dependent on distances, size of the operating area and the spatial distribution of missions within it. On the contrary, response time or duration in proportion to the service area, which is defined using a standardized method, would provide a result that could also be used for quality comparison.

We found that setting a cut point percentile significantly affects the results of this method. Based on this data, a 95% cut point appears to be the most descriptive, as it seems to be in line with the everyday experience of the area where the unit actually operates. On the other hand, 95% is also widely used in statistics as an indicator of high confidence, for example, in confidence limits.

Using the 95% cut point for all Finnish HEMS bases resulted in creating service areas that are partly overlapping, meaning that the same geographical area and its population belong to several service areas. It can be assumed that HEMS is more likely to be dispatched for missions that are located within these overlapping areas as they are reachable from multiple HEMS bases. If the service area (by km^2^ or population) is used as a QI, these factors might have to be taken into consideration. However, we have tried to create a method simple enough to implement everywhere; even if not perfect, any standardized method based on data will give more accurate and comparable results than areas defined by administrative boundaries.

While spatial analysis in EMS has been mainstream for decades, only few studies have been conducted on HEMS operations [7]. Discussion on the cost and benefits of HEMS demands accurate and comparable analysis methods. The proposed method, and the service areas defined by it, could also be used for planning new HEMS units or for developing HEMS systems; a previous study shows that using population density to define optimal base locations is not recommended, as the recommended location may not correspond to incident frequency [8].

The method described in this study was used to determine the service areas of HEMS units in Finland. In the future, however, the method should be tested with more units and in different environments to confirm its usefulness for international comparison of HEMS services and systems. The method is very straightforward, requiring only mission locations as input data, and it can be done using basic statistical and GIS software and even with freely available open source tools. Thus, it is simple to repeat for any HEMS unit for which mission location data is available.

### Limitations

The current method does not take into consideration spatial variation within the areas. Thus, to further develop the method and improve the comparability of services areas, water bodies and wilderness areas without inhabitation and road access should be excluded from the convex hull area during additional tests.

## Conclusion

Using administrative boundaries to define the service area for a HEMS unit may produce results that differ significantly from actual operations. Using historical mission data to create a convex hull that incorporates mission locations could offer a standardized and comparable solution for determining actual HEMS unit service areas that can be used for statistical comparison, quality measurement and system development.

## Data Availability

The data are available from the authors upon reasonable request.
